# Contemporary incidence and risk factors of post transplant Erythrocytosis in deceased donor kidney transplantation

**DOI:** 10.1186/s12882-021-02231-2

**Published:** 2021-01-12

**Authors:** Sami Alasfar, Isaac E. Hall, Sherry G. Mansour, Yaqi Jia, Heather R. Thiessen-Philbrook, Francis L. Weng, Pooja Singh, Bernd Schröppel, Thangamani Muthukumar, Sumit Mohan, Rubab F. Malik, Meera N. Harhay, Mona D. Doshi, Enver Akalin, Jonathan S. Bromberg, Daniel C. Brennan, Peter P. Reese, Chirag R. Parikh

**Affiliations:** 1 Division of Nephrology, Johns Hopkins University School of Medicine, Baltimore, MD USA; 2 Johns Hopkins School of Medicine, 1830 E. Monument St., Suite 416, Baltimore, MD 21287 USA; 3 Department of Internal Medicine, Division of Nephrology & Hypertension, University of Utah School of Medicine, Salt Lake City, UT USA; 4 Program of Applied Translational Research, Yale University School of Medicine, New Haven, CT USA; 5 Department of Internal Medicine, Section of Nephrology, Yale University School of Medicine, New Haven, CT USA; 6 Saint Barnabas Medical Center, RWJ Barnabas Health, Livingston, NJ USA; 7 Department of Medicine, Division of Nephrology, Sidney Kimmel Medical College, Thomas Jefferson University Hospital, Philadelphia, PA USA; 8 Section of Nephrology, University of Ulm, Ulm, Germany; 9 Department of Medicine, Division of Nephrology and Hypertension, New York Presbyterian Hospital-Weill Cornell Medical Center, New York, NY USA; 10 Department of Transplantation Medicine, New York Presbyterian Hospital-Weill Cornell Medical Center, New York, NY USA; 11 Department of Epidemiology, Columbia University Mailman School of Public Health, New York, NY USA; 12 Department of Medicine, Division of Nephrology, Columbia University Vagelos College of Physicians & Surgeons, New York, NY USA; 13 Department of Medicine, Drexel University College of Medicine, Philadelphia, PA USA; 14 Department of Epidemiology and Biostatistics, Drexel University Dornsife School of Public Health, Philadelphia, PA USA; 15 Department of Internal Medicine, Division of Nephrology, University of Michigan Medical School, Ann Arbor, MI USA; 16 Kidney Transplant Program, Montefiore Medical Center, Albert Einstein College of Medicine, Bronx, NY USA; 17 Department of Surgery, Division of Transplantation, University of Maryland School of Medicine, Baltimore, MD USA; 18 Department of Microbiology and Immunology, University of Maryland School of Medicine, Baltimore, MD USA; 19 Department of Medicine, Renal-Electrolyte and Hypertension Division, University of Pennsylvania Perelman School of Medicine, Philadelphia, PA USA; 20 Department of Biostatistics, Epidemiology & Informatics, University of Pennsylvania Perelman School of Medicine, Philadelphia, PA USA; 21 Department of Medical Ethics and Health Policy, University of Pennsylvania Perelman School of Medicine, Philadelphia, PA USA

**Keywords:** Erythrocytosis, Kidney transplant, Hemoglobin, KDPI

## Abstract

**Background:**

Post-Transplant erythrocytosis (PTE) has not been studied in large recent cohorts. In this study, we evaluated the incidence, risk factors, and outcome of PTE with current transplant practices using the present World Health Organization criteria to define erythrocytosis. We also tested the hypothesis that the risk of PTE is greater with higher-quality kidneys.

**Methods:**

We utilized the Deceased Donor Study which is an ongoing, multicenter, observational study of deceased donors and their kidney recipients that were transplanted between 2010 and 2013 across 13 centers. Eryrthocytosis is defined by hemoglobin> 16.5 g/dL in men and> 16 g/dL in women. Kidney quality is measured by Kidney Donor Profile Index (KDPI).

**Results:**

Of the 1123 recipients qualified to be in this study, PTE was observed at a median of 18 months in 75 (6.6%) recipients. Compared to recipients without PTE, those with PTE were younger [mean 48±11 vs 54±13 years, *p* < 0.001], more likely to have polycystic kidney disease [17% vs 6%, *p* < 0.001], have received kidneys from younger donors [36 ±13 vs 41±15 years], and be on RAAS inhibitors [35% vs 22%, *p* < 0.001]. Recipients with PTE were less likely to have received kidneys from donors with hypertension [16% vs 32%, *p* = 0.004], diabetes [1% vs 11%, *p* = 0.008], and cerebrovascular event (24% vs 36%, *p* = 0.036). Higher KDPI was associated with decreased PTE risk [HR 0.98 (95% CI: 0.97–0.99)]. Over 60 months of follow-up, only 17 (36%) recipients had sustained PTE. There was no association between PTE and graft failure or mortality,

**Conclusions:**

The incidence of PTE was low in our study and PTE resolved in majority of patients. Lower KDPI increases risk of PTE. The underutilization of RAAS inhibitors in PTE patients raises the possibility of under-recognition of this phenomenon and should be explored in future studies.

**Supplementary Information:**

The online version contains supplementary material available at 10.1186/s12882-021-02231-2.

## Background

Post-transplant erythrocytosis (PTE) has a reported incidence between 8 and 20% and is a potentially serious complication following kidney transplantation (KT) with increased morbidity and risk for thromboembolic events [1–7]. Several studies have assessed the incidence, risk factors, and impact of PTE on outcomes following KT [1–10]; however, reports about PTE in the background of modern immunosuppression are lacking. In addition, previously published studies have several limitations.

Most of these studies lacked standardized definition of PTE. Some studies used cut off values of hematocrit (50 to 53.5%), or hemoglobin (16.5–18 g/dL), whereas others used a cut off value of actual red cell mass [1–5, 7–9, 11–13]. Despite the variation in normal hemoglobin values between men and women, some studies and guidelines, including KDIGO 2009 guidelines, used the same threshold to define PTE in both sexes while others used separate sex-based cutoffs [14]. The duration of erythrocytosis in prior studies has also been variable with some including all patients above the cutoff value at any time after transplantation, whereas others required persistence of PTE beyond a set period of time. Most of the prior studies have been from single centers and included patients who were transplanted before the adoption of T-cell depleting induction immunosuppression and mycophenolate for maintenance. These newer agents can cause significant bone marrow suppression and may potentially reduce the incidence of PTE. In addition, some studies have suggested that PTE is more common in KT recipients with well-preserved renal allograft function [6, 9] and in recipients of simultaneous pancreas and KT, which could be explained by the favorable donor quality that these recipients enjoy [13].

In 2016, the World Health Organization (WHO) revised the cutoff values for diagnosing erythrocytosis to include hemoglobin > 16.5 g/dL in men and > 16 g/dL in women, hematocrit > 49% in men and > 48% in women, or red cell mass > 25% above the mean normal predicted value [15, 16]. These are lower values than previously used to define PTE. As there are no large multicenter studies of PTE in the modern era of immunosuppression, we utilized the ongoing Deceased Donor Study (DDS) cohort to evaluate the incidence and risk factors of PTE with current transplant practices and using the present WHO approved definition. We also tested the hypothesis that the risk of PTE is greater with higher-quality kidneys [i.e., lower kidney donor profile index (KDPI)] in deceased-donor KT and assessed the impact of PTE on patient and allograft outcome.

## Methods

### Study cohort

The study cohort is derived from the Deceased Donor Study (DDS), which is an ongoing multicenter, observational, cohort of deceased donors and their kidney recipients [17–20]. Briefly, five organ procurement organizations (OPOs) enrolled donors between May 2010 and December 2013 and followed their own protocols for research authorization and donor management. Clinical variables were abstracted from OPO charts. We performed chart reviews for the subset of recipients at 13 participating transplant centers in our study network who received kidneys from enrolled donors. Trained site coordinators reviewed medical records and recorded detailed recipient characteristics, treatments, and outcomes. Data including hemoglobin measurements were abstracted at month 3, 6, 12, 18, 24, 30, 36, 48, and 60. Study staff at the data-coordinating center validated abstracted data to confirm data quality and accuracy (Supplementary Appendix A). Data for all kidney recipients and donors were also obtained from the Organ Procurement and Transplantation Network (OPTN). The OPTN data system includes data on all donors, wait-listed candidates, and transplant recipients in the United States, submitted by the members of the OPTN, and is described more fully elsewhere [21]. The Health Resources and Services Administration, US Department of Health and Human Services provides oversight of the activities of the OPTN. The current analyses are based on OPTN data as of December 31, 2018. The OPO scientific review committees and the institutional review boards for participating investigators approved the study. The methods and conduct of this research study were consistent with the Principles of the Declaration of Istanbul as outlined in the Declaration of Istanbul on Organ Trafficking and Transplant Tourism.

### Study variables

PTE was defined as having at least one measurement of hemoglobin > 16.5 g/dL in men and > 16 g/dL in women during the 5 years follow-up after transplant. Persistent PTE was defined as having at least two qualifying measurements of hemoglobin. Every recipient had hemoglobin abstracted from clinical charts at each time point (3, 6, 12, 18, 24, 30, 36, 48, 60 months) regardless whether the patient qualifies for PTE or not, eliminating any lead-time bias. Diagnosis of PTE was based on laboratory criteria and not based on documentation of PTE diagnosis in clinical chart. Possible causes of secondary erythrocytosis were not available in the abstracted data. Baseline characteristics were collected at the time of transplant per the DDS protocol. The primary outcome of interest is PTE up to 5 years after transplant and the time to first diagnosis of PTE. The secondary outcomes include death-censored graft failure, death only, or all-cause graft failure with median post-transplant follow-up of 4 (IQR = 3,5) years. Death censored graft failure was defined as primary non-function of kidney after transplant, re-transplantation, or return to dialysis during follow-up time before death. Death only was defined as all-cause mortality before graft failure. All-cause graft failure was defined as all-cause mortality, primary non-function, return to dialysis, or re-transplantation.

We used KDPI as the primary exposure. KDPI is derived from the kidney donor risk index (KDRI), which is calculated using the 10-variable equation generated by Rao, et al. [22]. The variables are comprised by the following donor characteristics: age, height, weight, ethnicity, history of hypertension, history of diabetes, cause of death, serum creatinine, hepatitis c virus sero-status from serological or nucleic acid testing, and donation after circulatory death (DCD) status. The KDPI represents a mapping of the KDRI, a measure of relative risk of allograft failure, to a cumulative percentage scale [23]. We calculated the KDPI relative to all deceased donors in the OPTN database in 2010.

### Statistical analyses

We reported descriptive statistics as means (standard deviation) or medians (inter-quartile range) for continuous variables, and frequencies (percentages) for categorical variables. Continuous variables were compared with Wilcoxon rank sum or Kruskal-Wallis tests, and categorical variables were compared with chi-square tests or Fisher exact tests. Cox proportional hazards models were used to evaluate the association between KDPI and the outcome of PTE. We accounted for the cluster effect of paired kidneys from the same donor using robust sandwich estimates We used Kolmogorov-type supremum tests to evaluate proportional hazards assumptions [24]. We also explored the association of PTE with each secondary outcomes using time-varying covariate Cox model [24]. For the time varying analysis, in recipients that developed PTE, the time from transplant to PTE diagnosis contributes to the non-event. The time from PTE to last follow-up (5 years or death) contributes as an event. The models adjusted for donor Kidney Donor Profile Index (KDPI), cold ischemia time, transplant center, and the following recipient variables: age (years), black race, sex, previous KT, number of human leukocytes antigen (HLA) mismatches, panel reactive antibody (%), body mass index (BMI) (kg/m^2^), and preemptive transplant. We conducted a complete case analysis because < 2% of data for all covariates was missing. SAS 9.4 software for Windows (SAS Institute, Cary, NC) was used for all analyses. All statistical tests and confidence intervals were 2-sided with a significance level of 0.05.

## Results

### Clinical characteristics

Fourteen recipients were excluded from the 1137 due to missing hemoglobin or KDPI data, leaving the study cohort of 1123 patients. The median follow-up time was 60 (36, 60) months. PTE was observed in 75 (6.6%) patients and the median time to development of PTE was 18 months (18, 24). Mean peak hemoglobin in patients with PTE was 17.12 ± 0.77 compared to 13.32 ±1.79 in patients without PTE (*p* < 0.001).

Table 1 shows clinical characteristics of recipients with and without PTE as well as their donors.

**Table 1:** Clinical characteristics of recipients with and without Post Transplant Erythrocytosis

Clinical Variable		ALL (***n*** = 1123)	No PTE (***n*** = 1048)	PTE (***n*** = 75)	***P*** value
**Recipients Characteristics**					
Mean age (Years)		53.6 (13.2)	54.0 (13.2)	48.3 (11.1)	< 0.001
African American race		524 (47%)	496 (47%)	28 (37%)	0.09
Mean BMI (kg/m2)		28.3 (5.6)	28.4 (5.6)	27.2 (5.5)	0.06
Gender (Male)		687 (61%)	628 (60%)	59 (79%)	0.001
Cause of ESRD	Diabetes	355 (32%)	330 (31%)	25 (33%)	< 0.001
	Hypertension	313 (28%)	294 (28%)	19 (25%)	
	Glomerulonephritis	182 (16%)	171 (16%)	11 (15%)	
	PKD	67 (6%)	54 (6%)	13 (17%)	
	Graft failure	91 (8%)	87 (8%)	4 (5%)	
	Other or unknown	115 (6%)	54 (5%)	13 (17%)	
Mean dialysis vintage (Months)^a^		53.4 (43.5)	53.6 (44.0)	51.2 (35.9)	0.86
History of prior KT		158 (14%)	151 (14%)	7 (9%)	0.22
Mean number of Human Leukocyte Antigen (HLA) mismatches (A,B,DR)		4.37 (1.33)	4.38 (1.34)	4.21 (1.08)	0.07
Mean calculated PRA		21.0 (35)	21.5 (35.3)	14.2 (29.1)	0.14
**Donor Characteristics**					
Mean age (Years)		41.5 (15.3)	41.9 (15.3)	36.32 (13.6)	0.002
African American race		179 (16%)	167 (16%)	12 (16%)	0.98
Mean height (cm)		170.7 (11.1)	170.5 (11.2)	173.9 (9.7)	0.02
Mean weight (kg)		82.5 (22.3)	82.5 (22.4)	82.1 (20.0)	0.76
History of diabetes		117 (10%)	116 (11%)	1 (1%)	0.008
History of hypertension		347 (31%)	335 (32%)	12 (16%)	0.004
Donation after circulatory death (DCD)		215 (19%)	199 (19%)	16 (21%)	0.61
History of hepatitis C		30 (3%)	26 (2%)	4 (5%)	0.13
Cerebrovascular death		395 (35%)	377 (36%)	18 (24%)	0.03
Mean terminal serum creatinine (mg/dL)		1.21 (0.93)	1.2 (0.9)	1.31 (1.37)	0.82

Continuous variables: Wilcoxon rank sum or Kruskal-Wallis tests, and categorical variables were compared with chi-square tests or Fisher exact tests

*BMI* Body Mass Index, *PKD* Polycystic Kidney Disease, *ESRD* End Stage Renal Disease

^a^For non-preemptive KT

Compared to recipients without PTE, those with PTE were younger [mean age 48 (11.2) vs 54 (13.3) years, *p* < 0.001], and more likely to have polycystic kidney disease (PKD) as the cause of their end stage renal disease [17% versus 6%, *p* < 0.001].

### Immunosuppression regimen and other drugs

Anti-thymocyte globulin [rabbit] and basiliximab were used for induction immunosuppression in 928 (83%) and 163 (15%) of recipients, respectively. Triple maintenance immunosuppression regimen consisting of a calcineurin inhibitor, mycopthenolate and prednisone was utilized in 82% of recipients. There was no difference in the choice of induction or maintenance immunosuppression at discharge, 12 months, and 24 months after transplant between those with and without PTE (Table 2).

**Table 2:** Comparison of choice & dose of immunosuppression regimen and use of RAAS inhibitors between subjects with and without Post Transplant Erythrocytosis

Medication	ALL (***n*** = 1123)	No PTE (***n*** = 1048)	PTE (***n*** = 75)	***P***-Value
**Induction Immunosuppression**				
Anti-Thymocyte Globulin	928 (83%)	861 (82%)	67 (89%)	0.27
Basiliximab	163 (15%)	154 (15%)	9 (12%)	0.72
Alemtuzumab	34 (3%)	31 (3%)	3 (4%)	0.73
Rituximab	16 (1%)	15 (1%)	1 (1%)	0.86
**Maintenance Immunosuppression at Discharge**				
Triple regimen with CNI, mycophenolate, and prednisone	918 (82%)	853 (81%)	65 (87%)	0.25
Tacrolimus	1075 (96%)	1000 (96%)	75 (100%)	0.17
Cyclosporine	17 (2%)	17 (2%)	0 (0%)	0.41
Mycophenolate	1086 (97%)	1014 (97%)	72 (96%)	0.52
Prednisone	956 (85%)	888 (85%)	68 (91%)	0.16
Others (Sirolimus or Everolimus or Belatacept)	6 (1%)	6 (1%)	0 (0%)	0.51
**Maintenance Immunosuppression at Follow-up up to 1 year**				
Mycophenolate	1048 (93%)	979 (93%)	69 (92%)	0.63
Average daily mycophenolate dose (mg)	1185.9 (480.3)	1184.5 (482.9)	1205.9 (446.1)	0.86
Tacrolimus	1075 (96%)	1000 (95%)	75 (100%)	0.05
Average daily tacrolimus dose (mg)	8.0 (4.8)	8.1 (4.9)	6.6 (4.0)	0.01
Average daily tacrolimus dose (mg/kg)	0.1 (0.07)	0.11 (0.64)	0.09 (0.06)	0.03
Cyclosporine	42 (4%)	42 (4%)	0 (0%)	0.07
Prednisone	953 (85%)	886 (85%)	67 (89%)	0.26
**Maintenance Immunosuppression at Follow-up up to 2 years**				
Mycophenolate	1058 (94%)	988 (94%)	70 (93%)	0.73
Average daily mycophenolate dose (mg)	1145.78 (435.04)	1143.26 (433.98)	1181.31 (451.47)	0.71
Tacrolimus	1078 (96%)	1003 (96%)	75 (100%)	0.06
Average daily tacrolimus dose (mg)	8.12 (22.29)	8.28 (3.09)	6.04 (3.45)	0.007
Average daily tacrolimus dose (mg/kg)	0.11 (0.28)	0.11 (0.29)	0.08 (0.05)	0.01
Cyclosporine	49 (4%)	48 (5%)	1 (1%)	0.18
Prednisone	963 (86%)	894 (85%)	69 (92%)	0.10
**Use of RAAS Inhibitors**				
At 3 months	62 (6%)	57 (6%)	5 (7%)	0.62
At 6 months	90 (9%)	80 (8%)	10 (14%)	0.11
Anytime within 12 months	182 (16%)	164 (16%)	18 (24%)	0.05
At 18 months	171 (18%)	154 (17%)	17 (25%)	0.11
At 24 months	181 (20%)	162 (19%)	19 (27%)	0.05
Anytime within 24 months	257 (23%)	231 (22%)	26 (35%)	0.01

Continuous variables: Wilcoxon rank sum or Kruskal-Wallis tests, and categorical variables were compared with chi-square tests or Fisher exact tests

*CNI* Calcineurin inhibitor

The average daily mycophenolate dose in the first 12 and 24 months following KT between PTE and non-PTE groups was similar [1206 (446) versus 1185 (483) mg, *p* = 0.86]. However, the average daily dose of tacrolimus was lower in the PTE group at 12 months [6.7 (4.0) versus 8.1 (4.9) mg, *p* = 0.01] and at 24 months [6.0 (3.5) versus 8.3 (3.1) mg, *p* = 0.007]. Renin-angiotensin-aldosterone system (RAAS) inhibitors were prescribed in 182 (16%) and 257 (23%) of the recipients in this cohort within 12 and 24 months following KT respectively. Within the first 12 months, 24% of those with PTE were on RAAS inhibitors during at least one follow-up visit compared to 16% of those without PTE (*p* = 0.05). Within the first 24 months, 35% of recipients with PTE were on RAAS inhibitors at some point compared to 22% of those without PTE (*p* = 0.01). Regarding the timing of starting RAAS inhibitors, 31 (41%) of patients with PTE were started on RAAS inhibitors after meeting criteria for PTE and 10 (13%) were already on RAAS inhibition at the time they met criteria for PTE. This leaves 34 (45%) patients with PTE who did not receive any RAAS inhibitors despite meeting criteria for PTE.

### KDPI and risk of PTE

**Fig. 1:**
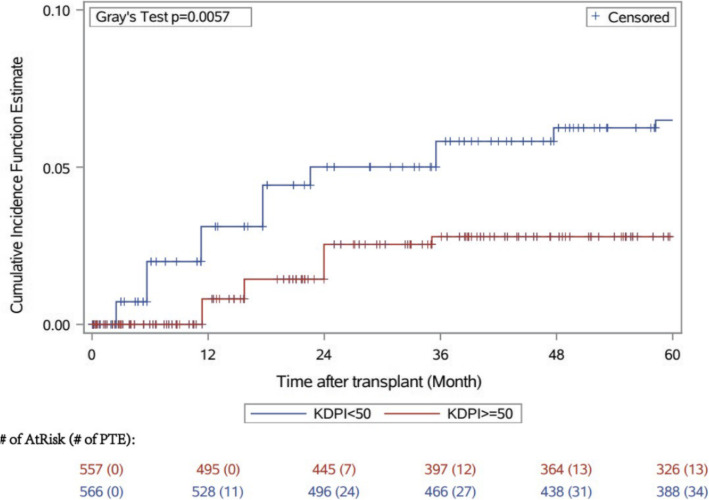
Cumulative incidence of Post Transplant Erythrocytosis by Kidney Donor Profile Index category [< 50 (n 566), >=50 (n 557)]

Figure 1 demonstrates the cumulative incidence of PTE by median KDPI category (< 50 versus ≥50) in the post-transplant period.

**Fig. 2:**
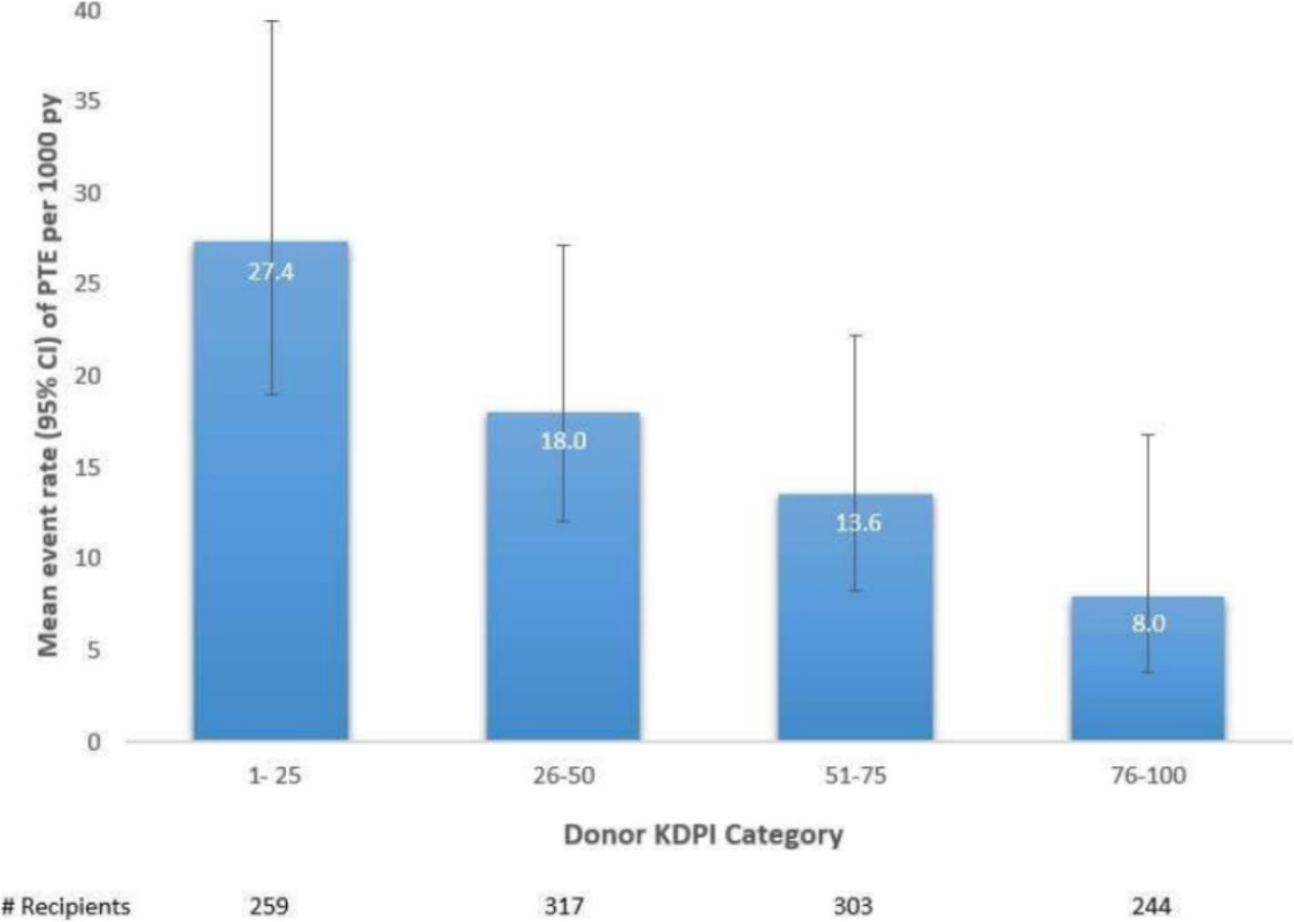
Event rate of Post Transplant Erythrocytosis by Kidney Donor Profile Index categories

Recipients of kidneys with KDPI ≥50% were less likely to develop PTE [HR 0.48 (0.29–0.78)]. We also observed a continuum of risk across the KDPI score such that, higher KDPI was associated with lower risk of PTE [HR 0.98 (0.97–0.99)] (Fig. 2).

Among components of KDPI, donor age, height, history of diabetes, history of hypertension, and cerebrovascular death as the cause of death were significantly different between PTE and non-PTE groups. Those with PTE were more likely to have received kidneys from donors with younger age and taller stature, and were less likely to have received kidneys from donors with history of hypertension, history of diabetes, and death from cardiovascular event. There was no association for donor race, weight, DCD status, history of hepatitis C, or terminal serum creatinine with recipient risk of PTE (Table 1).

### PTE and kidney transplant recipient outcomes

There were 4 (5%) deaths and 8 (11%) graft losses in recipients with PTE compared to 103 (10%) and 228 (22%) respectively in the non-PTE group. PTE was not associated with increased risk of death [unadjusted HR 0.88 (0.32, 2.41)], all cause allograft failure [unadjusted HR 0.81 (0.40, 1.64)], or death censored allograft failure [unadjusted HR 0.72 (0.27, 1.98)] over the 5 year follow up period (Table 3). As part of sensitivity analysis, we repeated this analysis by excluding the deaths and graft failure in the first 18 months during the observation period of development of PTE. The association of PTE with recipient outcomes was essentially unchanged (Supplementary Table 1S).

**Table 3:** Association of Post Transplant Erythrocytosis and patient & allograft survival

Outcome	Exposure	Number of event (%)	Mean event rate per 1000 patient year (95% CI)	Unadjusted Hazard Ratio (95% CI)	Adjusted 1 Hazard Ratio (95% CI)	Adjusted 2 Hazard Ratio (95% CI)
Death	No PTE (*n* = 1048)	103 (10%)	23.5 (19.4, 28.5)	1 (ref)	1 (ref)	1 (ref)
	PTE (*n* = 75)	4 (5%)	18.4 (6.9, 48.9)	0.88 (0.32, 2.41)	1.01 (0.50, 2.07)	1.07 (0.52, 2.20)
All-cause graft failure	No PTE (*n* = 1048)	228^a^ (22%)	52.0 (45.6, 59.2)	1 (ref)	1 (ref)	1 (ref)
	PTE (*n* = 75)	8 (11%)	36.7 (18.4, 73.4)	0.81 (0.40, 1.64)	1.07 (0.39, 2.94)	1.29 (0.46, 3.63)
Death-censored graft failure	No PTE (*n* = 1048)	131 (12%)	29.9 (25.2, 35.4)	1 (ref)	1 (ref)	1 (ref)
	PTE (*n* = 75)	4 (5%)	18.4 (6.9, 48.9)	0.72 (0.27, 1.98)	0.94 (0.35, 2.60)	0.94 (0.34, 2.60)

Adjusted 1 is adjusted for donor Kidney Donor Profile Index (KDPI)

Adjusted 2 is adjusted for donor KDPI, cold ischemia time and the following recipient variables: age (years), black race, sex, previous kidney transplant, number of human leukocyte antigen mismatches, panel reactive antibody (%), body mass index (kg/m2), preemptive transplant and transplant center

^a^There were 6 recipients who developed graft failure and then died. This explains the difference between the number of all-cause graft failure (228) and the sum of numbers of death (103) and death-censored graft failure (131)

There was no difference in the rate of hospitalization for any cause, hospitalization due to cardiac event, or acute rejection between the two groups (Table 4).

**Table 4:** Association of Post Transplant Erythrocytosis and selected Kidney Transplant outcomes

Follow-up Point	all (***n*** = 1123)	No PTE (***n*** = 1048)	PTE (***n*** = 75)	***P***-value
**Cumulative Hospitalizations**				
6 months	602 (54%)	568 (54%)	34 (45%)	0.70
12 months	682 (61%)	641 (61%)	41 (55%)	0.26
18 months	718 (64%)	674 (64%)	44 (59%)	0.32
24 months	743 (66%)	698 (67%)	45 (60%)	0.24
**Cumulative Hospitalizations due to Cardiovascular Event**				
6 months	22 (2%)	22 (2%)	0 (0%)	0.20
12 months	33 (3%)	32 (3%)	1 (1%)	0.39
18 months	41 (4%)	39 (4%)	2 (3%)	0.63
24 months	50 (4%)	48 (5%)	2 (3%)	0.43
**Cumulative Episodes of Rejection**				
6 months	93 (8%)	90 (9%)	3 (4%)	0.16
12 months	118 (11%)	113 (11%)	5 (7%)	0.26
18 months	133 (12%)	128 (12%)	5 (7%)	0.15
24 months	144 (13%)	139 (13%)	5 (7%)	0.09
**Estimated GFR**				
6 months	51.4 (20.4)	50.81 (20.43)	60 (18.17)	< 0.001
12 months	52.3 (20.4)	51.67 (20.44)	61.85 (18.57)	< 0.001
18 months	52.8 (20.3)	52.17 (20.34)	62.04 (17.81)	< 0.001
24 months	52.8 (21.3)	51.99 (21.21)	62.86 (20.37)	< 0.001

Continuous variables: Wilcoxon rank sum or Kruskal-Wallis tests, and categorical variables were compared with chi-square tests or Fisher exact tests

*GFR* Glomerular filtration rate

Recipients with PTE achieved higher estimated glomerular filtration rate (eGFR) compared to those without PTE at all points of follow-up within the first 24 months.

Only 52% (31 out of 59) of men and 18% (3 out of 16) of women with PTE were observed to have persistent PTE. There was no difference in the use of RAAS inhibitors between those with and without persistent PTE [21% versus 18% at 12 months (p 0.64) and 35% versus 34% at 24 months (p 0.91)].

## Discussion

This large multicenter study provides significant updates to the existing knowledge in PTE. We utilized longitudinally collected data over a long follow-up period to re-examine risk factors and outcomes of PTE in the era of modern immunosuppression, and using standardized definitions with consideration for gender differences.

Earlier studies established PTE as an erythropoietin-dependent phenomenon [7, 11, 25, 26]. One of the key findings of our study is the association between deceased donor kidney quality, measured by KDPI, and risk of PTE, such that, recipients with best quality kidneys had the highest risk of developing PTE. This inverse association of KDPI with PTE was present across the continuum of kidney quality score. In addition, we observed relationship between by traditional risk factors for chronic kidney disease (CKD) in the donor, which were age, diabetes, hypertension, and cerebrovascular disease with reduced occurrence of PTE. These findings suggest that clinical risk factors may lead to reduced sensitivity of erythropoietin-producing fibroblasts in the renal cortex before developing clinically evident CKD.

RAAS inhibitors have been shown to be effective in the treatment of PTE and therefore are used as first-line therapy [7, 10, 27–31]. Although our study showed that recipients with PTE were more likely to be on RAAS inhibitors, these agents remained underutilized as a significant proportion of patients with PTE were not receiving them. This could be due to under recognition of this phenomenon by using older non-standardized definitions, non-familiarity with the new definition, or due to contraindications for using RAAS inhibitors. It is worth mentioning that the criteria for erythrocytosis was revised in 2016, several years after DDS cohort was established. Interestingly, only about half of the patients with PTE continued to have persistent elevation in hemoglobin over the follow-up period, and there was no difference in RAAS inhibitors use between the persistent and non-persistent groups.

Concerning PTE impact on transplant outcomes, our study showed that those with PTE did not achieve superior patient or allograft survival over a follow-up period of 5 years despite having better kidney quality, therefore, arguing the possibility that PTE may mitigate the expected favorable outcomes of receiving a higher-quality kidney. It is worth recalling here that a previous study by Kiberd et al. showed that recipients with PTE achieved superior overall survival but had inferior renal allograft survival [5]. We did not observe inferior renal allograft outcome in our study, and one may hypothesize that the advancement in immunosuppression and medical management of transplant recipients have outweighed the possible negative impact of PTE. PTE has been traditionally associated with increased risk of thromboembolic and cardiovascular events such as stroke and myocardial infarction. The reported incidence of these events is variable depending on the study and duration of follow-up, but it ranged between 10 and 40% [1, 9, 32, 33]. Nonetheless, our study did not show increased risk for mortality, overall hospitalizations, or hospitalization due to cardiovascular events.

Our study is the first to assess the natural course of PTE using the updated WHO criteria to define erythrocytosis with lower hemoglobin thresholds in a multi-center seting [15]. Despite that, the incidence of PTE was only 6.6% in the first 5 years following KT, which is lower than previously reported. Earlier studies reported incidence rates between 8 and 20% [1, 2, 8, 9, 11, 28, 34]. Kiberd et al. compared the incidence of PTE between 1993 and 1996 and 1997–2005 and observed a declining incidence rate from 18.7 to 8.1%. This lower trend is more consistent with our study and could be explained by the adoption of more potent myelosuppressive agents such as anti-thymocyte globulin [rabbit] and mycophenolate for immunosuppression. However, we did not observe a difference in mycophenolate dose between PTE and non-PTE groups. In contrast, our study showed that patients with PTE received lower average daily doses of tacrolimus compared to those without PTE during follow-up. Anemia is a reported side effect of tacrolimus, and one may hypothesize that the widespread use of tacrolimus in the last two decades has contributed to the overall declining incidence of PTE. It is also possible that lack of live donor kidney transplant recipients contributed to the low incidence of PTE in our study although the association of PTE with receiving live donor kidneys has not been reported in prior studies.

Consistent with previous studies, we found an association between PTE and recipient sex, renal allograft function, and end-stage renal disease due to polycystic kidney disease [1, 2, 6, 9, 12, 34]. Our study also demonstrated an association of recipient age, race, and BMI with PTE. In contrast to original observations, we did not find an association between PTE and recipient history of diabetes [1], history of hypertension [1, 11], duration of dialysis [9], renal allograft rejection [1, 6], or use of mammalian target of rapamycin (mTOR) inhibitors [4]. We were unable to assess for potential relationships between PTE and retention of native kidneys [11], diuretic use [11], or tobacco use [1], which have been reported in previous studies.

Some limitations exist in the current study. All patients in this cohort were recipients of deceased-donor kidney transplants which limit generalizability to living donor kidney transplant recipients. We could not assess rates of certain PTE-related complications such as thromboembolic events because the occurrence of these events was not systematically collected during follow-up. For the same reason, the presence of secondary causes of PTE such as tobacco use, malignancy, lung disease, sleep apnea, and renal artery stenosis were not assessed. Other therapies that have been traditionally used as second line treatments for PTE such as therapeutic phlebotomy [35], and theophylline [36], as well as the presence of contraindications to using RAAS inhibitors were not evaluated.

In conclusion, our study demonstrated a low incidence of PTE in the current era of immunosuppression and an association between PTE and quality of donated kidneys. This association is driven by traditional risk factors for chronic kidney disease in the donor. Despite the low incidence, the underutilization of RAAS inhibitors in patients with PTE raises the possibility of under-recognition of this phenomenon. We could not demonstrate any associations between PTE and recipient outcomes, but lack of improved graft survival in recipient receiving higher quality kidneys is concerning. Our findings need to be confirmed in larger cohorts to allow for improved recognition and management of PTE.

## Supplementary Information

**Additional file 1: Table 1S**. Association of Post Transplant Erythrocytosis and Patient & Allograft survival After Exclusion of Events in First 18 Months

## Data Availability

All data generated or analyzed during this study are available from the corresponding author on reasonable request and will be saved and available for 5 years after publication.

## Abbreviations

BMI: Body Mass Index
DCD: Donation after Circulatory Death
DDS: Deceased Donor Study
CKD: Chronic Kidney Disease
eGFR: Estimated Glomerular Filtration Rate
ESRD: End Stage Renal Disease
HLA: Human Leukocytes Antigen
KDPI: Kidney Donor Profile Index
KDRI: Kidney Donor Risk Index
KT: Kidney Transplantation
mTOR: Mammalian Target of Rapamycin
OPO: Organ Procurement Organizations
OPTN: Organ Procurement and Transplantation Network
PKD: Polycystic Kidney Disease
PTE: Post Transplant Erythrocytosis
RAAS: Renin-Angiotensin-Aldosterone System
WHO: World Health Organization
