# Supplementary material for: Contemporary incidence and risk factors of post transplant Erythrocytosis in deceased donor kidney transplantation
Source: BMC Nephrol. 2021 Jan 12;22:26. doi: 10.1186/s12882-021-02231-2 (PMC7802150; doi:10.1186/s12882-021-02231-2)
Supplement: Supplementary file 1 — Additional file 1: Table 1S. Association of Post Transplant Erythrocytosis and Patient & Allograft survival After Exclusion of Events in First 18 Months [file 12882_2021_2231_MOESM1_ESM.pdf]

Title: Contemporary Incidence and Risk Factors of Post Transplant Erythrocytosis in Deceased Donor Kidney Transplantation

Sami Alasfar[1]
Isaac E. Hall, MD, MS[2]
Sherry G. Mansour, DO, MS[3,4]
Yaqi Jia, MPH[1]
Heather R. Thiessen-Philbrook, MMath[1]
Francis L. Weng, MD, MSCE[5]
Pooja Singh, MD[6]
Bernd Schröppel, MD[7]
Thangamani Muthukumar, MD[8,9]
Sumit Mohan, MD, MPH[10,11]
Rubab F Malik[1]
Meera N. Harhay, MD, MSCE[12,13]
Mona D. Doshi, MD[14]
Enver Akalin, MD[15]
Jonathan S. Bromberg, MD, PhD[16,17]
Daniel C. Brennan, MD[1]
Peter P. Reese, MD, MSCE[18-20]
Chirag R. Parikh, MD, PhD[1]

Affiliations:

1. Division of Nephrology, Johns Hopkins University School of Medicine, Baltimore, MD, USA
2. Department of Internal Medicine, Division of Nephrology & Hypertension, University of Utah School of Medicine, Salt Lake City, UT, USA
3. Program of Applied Translational Research, Yale University School of Medicine, New Haven, CT, USA
4. Department of Internal Medicine, Section of Nephrology, Yale University School of Medicine, New Haven, CT, USA
5. Saint Barnabas Medical Center, RWJ Barnabas Health, Livingston, NJ, USA
6. Department of Medicine, Division of Nephrology, Sidney Kimmel Medical College, Thomas Jefferson University Hospital, Philadelphia, PA, USA
7. Section of Nephrology, University of Ulm, Ulm, Germany
8. Department of Medicine, Division of Nephrology and Hypertension, New York Presbyterian Hospital-Weill Cornell Medical Center, New York, NY, USA
9. Department of Transplantation Medicine, New York Presbyterian Hospital-Weill Cornell Medical Center, New York, NY, USA
10. Department of Epidemiology, Columbia University Mailman School of Public Health, New York, NY, USA
11. Department of Medicine, Division of Nephrology, Columbia University Vagelos College of Physicians & Surgeons, New York, NY, USA
12. Department of Medicine, Drexel University College of Medicine, Philadelphia, PA, USA

[13] Department of Epidemiology and Biostatistics, Drexel University Dornsife School of Public Health, Philadelphia, PA, USA
[14] Department of Internal Medicine, Division of Nephrology, University of Michigan Medical School, Ann Arbor, MI, USA
[15] Kidney Transplant Program, Montefiore Medical Center, Albert Einstein College of Medicine, Bronx, NY, USA
[16] Department of Surgery, Division of Transplantation, University of Maryland School of Medicine, Baltimore, MD, USA
[17] Department of Microbiology and Immunology, University of Maryland School of Medicine, Baltimore, MD, USA
[18] Department of Medicine, Renal-Electrolyte and Hypertension Division, University of Pennsylvania Perelman School of Medicine, Philadelphia, PA, USA
[19] Department of Biostatistics, Epidemiology & Informatics, University of Pennsylvania Perelman School of Medicine, Philadelphia, PA, USA
[20] Department of Medical Ethics and Health Policy, University of Pennsylvania Perelman School of Medicine, Philadelphia, PA, USA

Corresponding Author:
Sami Alasfar, MD
Assistant Professor of Medicine
Johns Hopkins School of Medicine
1830 E. Monument St., Suite 416
Baltimore, MD 21287
Phone: (410) 955-5268
Fax: (410) 367-2259
salasfa1@jhmi.edu

Supplementary Appendix A: Data quality description

Trained study participants at each site recorded recipient data on standardized case report forms using a secure, online database. Outcomes were adjudicated by site investigators, and extensive data quality control checks were performed by study monitors from the data coordinating center (Yale University at the time of data abstraction, Johns Hopkins University at the time of data analysis). Study monitors validated data abstraction processes via secondary review of submitted paper charts and then continuously reviewed all data points following form completion by site coordinators to confirm data quality and accuracy.

This study utilized various methods such as rigorous study documentation, in-depth coordinator training, independent data monitoring, and principal investigator (PI) involvement to increase the quality of the data collected. A detailed protocol and a manual of operations were created to facilitate consistency in data collection practices across all participating research centers. Coordinators were required to attend two separate web-based trainings provided by a co-investigator, a study monitor (hired from the Yale Center for Clinical Investigation – separate from our department), and a project coordinator. The trainings demonstrated how to use the database (OnCore) and detailed explanation of important data variables and key practices to utilize during chart abstraction.

Research centers were all required to send the first five completed charts to the study monitor for data verification from source documents. All of the remaining charts were validated remotely by following defined guidelines for data quality developed by

the Yale Data Coordinating Center. Queries were used for quality control checks to identify potential data anomalies such as missing data or forms, out-of-range or erroneous data, and inconsistent data. The statistician also conducted separate back-end data checks. For instance, if the baseline form indicated a biopsy report was available at 3 month, the statistician would check to see if the biopsy details were provided in the 3-month follow-up form. If it was not, sites would be queried to enter the information. Participants who met the study stopping criteria were verified by each site PI. Monthly PI and coordinator calls were utilized to inform sites about timelines and any other updates that required their attention.

Table 1S. Association of Post Transplant Erythrocytosis and Patient & Allograft survival After Exclusion of Events in First 18 Months

Outcome	Exposure	Number of event (%)	Mean event rate per 1000 patient year (95% CI)	Unadjusted Hazard Ratio (95% CI)	Adjusted 1 Hazard Ratio (95% CI)	Adjusted 2 Hazard Ratio (95% CI)
Death	No PTE (n=936)	60 (6%)	13.9 (10.8, 17.8)	1 (ref)	1 (ref)	1 (ref)
	PTE (n=73)	4 (5%)	18.4 (6.9, 49.0)	0.98 (0.36, 2.71)	1.21 (0.44, 3.36)	1.47 (0.51, 4.22)
All-cause graft failure	No PTE (n=936)	131*(14%)	30.2 (25.5, 35.9)	1 (ref)	1 (ref)	1 (ref)
	PTE (n=73)	8 (11%)	36.8 (18.4, 73.6)	0.90 (0.44, 1.84)	1.10 (0.54, 2.26)	1.13 (0.54, 2.36)
Death-censored graft failure	No PTE (n=936)	72 (8%)	16.7(13.2, 20.9)	1 (ref)	1 (ref)	1 (ref)
	PTE (n=73)	4 (5%)	18.4 (6.9, 49.0)	0.82(0.30, 2.24)	1.01 (0.37, 2.78)	0.95 (0.34, 2.67)

1. This table patients with graft failure or death or lost to follow up within 18 months. As a result, 112 out of 1048 non PTE patients were excluded (97 due to death or GF, 15 due to lost to follow up), and 2 out of 75 PTE patients were excluded, both were due to lost to follow up.

2. Adjusted 1 is adjusted for donor Kidney Donor Profile Index (KDPI).

3. Adjusted 2 is adjusted for donor KDPI, cold ischemia time and the following recipient variables: age (years), black race, sex, previous kidney transplant, number of human leukocyte antigen mismatches, panel reactive antibody (%), body mass index (kg/m2), preemptive transplant and transplant center.

* There were 1 recipient who developed graft failure and then died. This explains the difference between the number of all-cause graft failure (131) and the sum of numbers of death (60) and death-censored graft failure (72).
